# Extreme temperature exposure increases the risk of preterm birth in women with abnormal pre-pregnancy body mass index: a cohort study in a southern province of China

**DOI:** 10.3389/fpubh.2023.1156880

**Published:** 2023-07-27

**Authors:** Jialing Qiu, Zhijiang Liang, Jing Yi, Lulu Xie, Qianqian Xiang, Xianqiong Luo, Qingguo Zhao

**Affiliations:** ^1^Department of Public Health, Guangdong Women and Children Hospital, Guangzhou, China; ^2^Department of Obstetrics, Guangdong Women and Children Hospital, Guangzhou, China; ^3^Department of Pediatric, Guangdong Women and Children Hospital, Guangzhou, China; ^4^Department of Public Health and Preventive Medicine, School of Medicine, Jinan University, Guangzhou, China; ^5^Department of Pediatric Endocrinology and Inherited Metabolic Diseases, Guangdong Women and Children Hospital, Guangzhou, China; ^6^Epidemiological Research Office of Key Laboratory of Male Reproduction and Genetics, Family Planning Research Institute of Guangdong Province, Guangzhou, China; ^7^Epidemiological Research Office of Key Laboratory of Male Reproduction and Genetics (National Health and Family Planning Commission), Guangdong Province Fertility Hospital, Guangzhou, China

**Keywords:** preterm birth, body mass index, ambient temperature, pre-pregnancy, cohort study, climate change

## Abstract

**Background:**

Prior literature has found that extreme temperature exposure is associated with preterm birth (PTB). However, current evidence provides heterogeneous conclusions, and data on extreme cold and across different pre-pregnancy body mass index (BMI) statuses are limited.

**Methods:**

We conducted a population-based retrospective cohort of 251,257 women between 2014 and 2017 in Guangdong, China, to evaluate whether the association between extreme temperature exposure and PTB varied in pre-pregnancy BMI status. Participants were divided into three categories based on pre-pregnancy BMI: underweight (BMI < 18.5 kg/m^2^), normal weight (18.5–23.9 kg/m^2^), overweight or obesity (≥ 24.0 kg/m^2^). We fitted Cox proportional hazards models to assess the association between daily mean temperature and PTB at each trimester for each BMI category separately. The hazard ratios (HRs) at the 5th and 95th percentiles of temperature (defined as low and high temperatures respectively) were provided using the median temperature at each trimester as a reference.

**Results:**

58,220 (23.2%) were underweight, and 27,865 (11.1%) were overweight or obese. Of the 251,257 women, 18,612 (7.41%) had PTB delivery. Both low-and high-temperature exposure increased the risk of PTB in the third trimester, while cold exposure mostly mitigated the risk for the first and second trimesters. The association with low temperature was the strongest in the third trimester, especially for underweight women (HR: 1.825; 95%CI: 1.529 ~ 2.179), while the association with high temperature was the strongest also in the third trimester, especially for obese or overweight women (HR:1.825; 95%CI:1.502 ~ 2.218). Furthermore, the attributable fractions of PTB risk in the third trimester were estimated as 5.59% (95% CI: 3.58, 7.98%) for cold exposure among underweight women and 3.31% (95% CI: 2.01, 4.88%) for hot exposure among overweight or obese women.

**Conclusion:**

Exposure to either low temperature in the third trimester or high temperature during pregnancy was associated with a higher risk of PTB. Moreover, pre-pregnancy BMI status might affect the susceptibility of pregnant women. Such findings would be useful to develop targeted measures for vulnerable populations.

## 1. Introduction

Preterm birth (PTB) is an important global maternal and child health problem, which causes a substantial burden to both the family and society. Data show that there are approximately 15 million preterm babies worldwide annually, estimating a PTB prevalence of about 11% (1). Complications of PTB are currently the leading killer among neonates and children under five, and those born preterm also have a high risk of short-and long-term morbidity (2, 3). China is a large country with the second-highest number of PTB globally, and its PTB rate has continued to increase in the past decades (4, 5). Moreover, with the implementation of the multiple-child policies, China’s maternal profile has undergone significant changes, such as increased maternal age and pregnancy complications, meaning more attention is required to reduce adverse birth outcomes in the future (6).

Recently, there has been increasing evidence suggesting a significant association between extreme temperature exposure and PTB. Biomedical studies have suggested that heat stress during pregnancy can affect maternal hormone secretion and alter fetal metabolism, thereby triggering preterm delivery (7–9). On the other hand, hypothermia exposure may also lead to increased blood vasoconstriction and hormone release, which also affect fetal development (10). Epidemiological studies demonstrated that exposure to high ambient temperatures was positively associated with PTB, while exposure to cold might either increase or decrease PTB (11). Other studies have shown that heat waves or cold spells significantly increased the risk of PTB (12). Overall, the current literature about extreme temperature exposure and PTB is concentrated in developed countries such as Europe and the United States. Moreover, the content of the research mainly focuses on the health impact of heat exposure, but insufficient attention is paid to low temperatures (13). Meanwhile, the conclusions between studies have yet to reach a consensus. More studies on regions with diverse climatic characters and population characteristics would be beneficial to reveal the association between extreme temperature and adverse pregnancy outcomes.

Individual maternal factors are also critical to PTB. Among them, maternal pre-pregnancy BMI is an important clinical indicator, and excessive obesity typically leads to obstetric complications and contributes to potential adverse pregnancy outcomes (14, 15). Specifically, research indicated that being underweight and overweight before pregnancy increased the risk of PTB (14–19). However, under the context of global warming and frequent extreme weather events, whether maternal issues of abnormal pre-pregnancy BMI would amplify the adverse health impact of environmental exposure, such as extreme temperature remains unclear. Women with poor baseline health status are particularly vulnerable to extreme ambient temperatures. Clarifying this would have implications for risk stratification and targeting subgroups in developing future interventions.

Guangdong, a subtropical province in China with the largest number of births nationally in 2021, provides a suitable place to explore extreme temperature exposure and adverse birth outcomes (20). Given the knowledge gap, this study aimed to investigate the risk of PTB induced by extreme temperature exposure by analyzing the data from a large-scale cohort study among reproductive-age women in Guangdong Province from 2014 to 2017. Specifically, we estimated the trimester effect of extreme heat and cold exposure. In addition, the difference in the associations among maternal BMI subgroups was identified.

## 2. Methods

### 2.1. Study design and participants

Women who participated in the National Free Preconception Health Examination Project (NFPHEP) and had successful pregnancies were recruited. NFPHEP is a national program initiated by the Chinese government for improving pregnancy outcomes. It provides free preconception care and follow-up to married couples who plan to conceive within the next 6 months. More details about the project design and implementation were provided in previous studies (21, 22).

In this study, we recruited 263,868 women from 21 cities in Guangdong Province from 2014 to 2017. Furthermore, the following cases were excluded: 1594 women with extreme birth weight (< 500 g or > 5,000 g), 3,582 women with multiple births, 230 women with chronic diseases (hepatitis B, thyroid disease, high blood pressure, diabetes, anemia, heart disease, epilepsy, chronic kidney disease, and tumor) before pregnancy, 249 women with abortion (spontaneous or induced), stillbirth, birth defects, 6,956 women with missing information on delivery mode, maternal age or pre-pregnancy BMI status. Therefore, the data of 251,257 women finally remained.

### 2.2. Baseline and follow-ups

At baseline, trained staff used the interviewer-administered questionnaire to collect the individual information of the participants, including socio-demographics (age, education, occupation, residential address), chronic disease history, pregnancy history, and lifestyle. Professional medical personnel provided physical examinations to the participants, including height and weight measurements. The pregnancy status of each participant was collected by telephone follow-ups every 2 months, and the time of her last menstrual period was recorded after the pregnancy was confirmed. Within 6 weeks of delivery, the participants were followed up to investigate whether they gave birth and the hospitals where they gave birth. Then the investigators extracted the related medical records from the corresponding hospitals and obtained the delivery-related information, including gestational age and delivery status. All data were uploaded to the online medical service information system supported by the National Institute of Family Planning. Quality control was conducted semiannually by the Quality Inspection Center of Guangdong Institute of Family Planning Science and Technology.

### 2.3. Air pollution and meteorological data

We collected air pollution data from the air quality monitoring stations across Guangdong province operated by the State Environmental Protection Administration of China, as described in our previous study (23). The daily concentrations of four typical pollutants, including ozone (O_3_), nitrogen dioxide (NO_2_), sulfur dioxide (SO_2_), and PM_2.5_ (particles with aerodynamic diameter < 2.5 μm), were obtained as the average of 24 hourly measurements. The concentration measurement was qualified according to the national standards and procedures proposed by the State Environmental Protection Administration of China (24). The pollutant concentrations of the stations in a district were averaged as district-specific exposure estimates, and then assigned to the woman living in the same district according to her residential address. We note that women without an air monitoring station in their districts were excluded from the analysis. All the participants lived in a district with a nearby air monitoring station at a radius of about 5 km. Meteorological information from the meteorological stations, including daily mean temperature(°C) and relative humidity (%), was obtained from National Weather Data Sharing System. Similarly, the district-specific exposures of daily mean temperature and relative humidity were estimated and matched to the participants using the same approach as that of air pollutants.

### 2.4. Definition of extreme temperature exposure and pre-pregnancy BMI categories

To identify the health impact of extreme temperature in different stages of pregnancy, we averaged the daily temperatures for each woman in the following three periods: the first trimester (0–12 weeks), the second trimester (13–27 weeks), and the third trimester (28 weeks or higher) (7, 25). For each trimester, high temperatures referred to temperatures above the 95th percentile, and low temperatures referred to temperatures below the 5th percentile, based on the distribution of daily mean air temperature exposures throughout this trimester.

Maternal pre-pregnancy body mass index (BMI, kg/m^2^) was measured as the ratio of maternal weight and height squared. According to Chinese standards, pre-pregnancy BMI was classified as: underweight (BMI <18.5), normal weight (18.5–23.9), overweight or obesity (≥24.0) (26).

### 2.5. Definition of PTB

The study outcome was PTB, defined as the birth at less than 37 gestational weeks. We calculated gestational age as the difference between the time of delivery and the last menstrual period.

### 2.6. Statistical analysis

We constructed Cox proportional hazards models to depict the risk of PTB from extreme temperature exposure, with PTB as the dependent variable and gestational weeks as the time variable. Considering the nonlinear relationship between meteorological factors and health outcomes, a natural cubic spline function with 3 degrees of freedom was adopted for the temperature at each trimester. The models were adjusted by a series of covariates, including the season of delivery, maternal age, delivery mode, newborn gender, history of adverse pregnancy outcomes, active smoking, husband smoke and alcohol drinking status during the early stage of pregnancy, relative humidity using a natural cubic spline with 3 degrees of freedom, and air pollutants. Models were constructed for PTB at each trimester for each BMI category separately. The risk of PTB at different temperatures was demonstrated by drawing exposure-response curves, and the hazard ratios (HRs) at the 5th and 95th percentiles of temperature (defined as low and high temperatures respectively) were provided using the median temperature at each trimester as a reference.

To demonstrate the public health burden of PTB due to extreme temperature exposure, we also calculated the attributable number and fraction of PTB (AN and AF respectively) due to the high-and low-temperature exposure, as described previously. This approach has been used widely in environmental research and is considered reasonable for estimating the health burden of temperature changes (27). The formula used was specified as:


$$\Delta y=y_{0}*\left(e^{\beta *\Delta x}-1\right)*\mathrm{Population}$$

where ∆*y* is the excess risk of PTB attributable to the extreme temperature exposure, $y_{0}$ is the baseline incidence rate of PTB, β is the coefficient derived from the HRs at the 5^th^ and 95^th^ percentiles of the temperature, ∆*x* is the estimated change in exposure, and Population is the exposed population. AF is calculated by dividing the total number of PTB by AN.

As for the sensitivity analyses, the robustness of the hazard models was determined by changing the degrees of freedom for mean temperature (degrees 2–4) and relative humidity (degrees 2–4). Model II was also performed by merely adjusting the season of delivery, mean of relative humidity and air pollutants. We used R software (version 3.5.2) for all the data analyses, and the test level α was set to 0.05 on both sides. In this study, the “survival” and “dlnm” packages were used to construct the Cox proportional hazards regression; the “smoothHR” and “splines” packages were used to perform point estimates and plot hazard ratio curves.

## 3. Results

Our final sample involved 251,257 women. Among them, 165,172 (65.7%) had normal weight, 58,220 (23.2%) had underweight, and 27,865 (11.1%) had overweight or obesity. The number of preterm births was 18,612 (7.41%). The baseline information of the pregnant women across different BMI levels is shown in Table 1. The mean birth weights for women with normal weight, underweight, and overweight or obesity, were 3182.1 ± 355.1, 3112.9 ± 342.8 and 3229.8 ± 384.1 years old, respectively. The mean maternal ages of women with normal weight, underweight, and overweight or obesity, were 27.3 ± 4.3, 25.7 ± 3.5 and 28.7 ± 4.9 years old, respectively. The distributions of baseline characteristics between women across different BMI levels were all statistically different (*p* < 0.05), except for alcohol drinking status during early pregnancy.

**Table 1:** Maternal baseline characteristics with respect to pre-pregnancy status of BMI.

Characteristics	Normal weight		Underweight		Overweight or Obesity	
	Preterm birth (*n* = 11,994)	Total births (*n* = 165,172)	Preterm birth (*n* = 4,373)	Total births (*n* = 58,220)	Preterm birth (*n* = 2,245)	Total births (*n* = 27,865)
Birth weight (grams, Mean (SD))	2939.0 (451.3)	3182.1 (355.1)	2893.5 (441.2)	3112.9 (342.8)	2950.0 (473.3)	3229.8 (384.1)
Maternal age (years, Mean (SD))	27.3 (4.6)	27.3 (4.3)	25.7 (3.7)	25.7 (3.5)	28.9 (5.2)	28.7 (4.9)
Maternal age, *n* (%)						
<25 years	3,610 (30.1)	46,755 (28.3)	1829 (41.8)	22,933 (39.4)	499 (22.2)	5,977 (21.5)
25 ~ 34 years	7,360 (61.4)	106,166 (64.3)	2,414 (55.2)	33,898 (58.2)	1,358 (60.5)	17,956 (64.4)
≥35 years	1,024 (8.5)	12,251 (7.4)	130 (3.0)	1,389 (2.4)	388 (17.3)	3,932 (14.1)
Delivery mode, *n* (%)						
Vaginal delivery	8,433 (70.3)	118,678 (71.9)	3,351 (76.6)	46,035 (79.1)	1,309 (58.3)	16,635 (59.7)
Cesarean section	3,561 (29.7)	46,494 (28.1)	1,022 (23.4)	12,185 (20.9)	936 (41.7)	11,230 (40.3)
Newborn sex, *n* (%)						
Male	6,735 (56.2)	86,751 (52.5)	2,475 (56.6)	30,202 (51.9)	1,272 (56.7)	14,756 (53.0)
Female	5,259 (43.8)	78,421 (47.5)	1898 (43.4)	28,018 (48.1)	973 (43.3)	13,109 (47.0)
Delivery season, *n* (%)						
Spring(March–May)	2,563 (21.4)	35,375 (21.4)	941 (21.5)	12,797 (22.0)	520 (23.2)	6,106 (21.9)
Summer(June–August)	2,974 (24.8)	40,131 (24.3)	1,107 (25.3)	13,735 (23.6)	549 (24.4)	6,936 (24.9)
Fall(September–November)	3,567 (29.7)	49,110 (29.7)	1,235 (28.2)	17,191 (29.5)	667 (29.7)	8,161 (29.3)
Winter(December–February)	2,890 (24.1)	40,556 (24.6)	1,090 (24.9)	14,497 (24.9)	509 (22.7)	6,662 (23.9)
History of adverse pregnancy outcomes, *n* (%)						
History of preterm	122 (1.0)	820 (0.5)	31 (0.7)	182 (0.3)	37 (1.6)	200 (0.7)
History of stillbirth	137 (1.1)	1816 (1.1)	32 (0.7)	445 (0.8)	59 (2.6)	450 (1.6)
History of miscarriage	495 (4.1)	6,644 (4.0)	148 (3.4)	1836 (3.2)	132 (5.9)	1,534 (5.5)
History of induced abortion	1,595 (13.3)	23,860 (14.4)	471 (10.8)	6,849 (11.8)	409 (18.2)	5,007 (18.0)
Lifestyle during early pregnancy ^a^, *n* (%)						
Active smoke	78/11751 (0.7)	879/161677 (0.5)	24/4286 (0.6)	386/57010 (0.7)	14/2200 (0.6)	155/27241 (0.6)
Husband smoke	2415/11713 (20.6)	33,546/161431 (20.8)	909/4249 (21.4)	12,595/56822 (22.2)	482/2201 (21.9)	6355/27193 (23.4)
Alcohol	116/11744 (1.0)	1672/161580 (1.0)	41/ 4,284 (1.0)	584/56980 (1.0)	26/2200 (1.2)	284/27230 (1.0)

a Denominators provided as missing data existed.

The meteorological and air pollution factors during the pregnancy are described in Table 2. The medians of daily mean temperature during Trimesters 1, 2 and 3 were 22.7, 24.5 and 24.7°C, respectively. The 5th percentile of daily mean temperatures (low temperatures) during Trimesters 1, 2 and 3 were 14.6, 15.7 and 15.3°C, respectively. The 95th percentile of daily mean temperatures (high temperatures) during Trimesters 1, 2 and 3 were 29.1, 29.0 and 29.2°C, respectively. The correlations were evaluated between daily mean temperature, relative humidity and air pollutants, as shown in Supplementary Tables S1. During the entire pregnancy, there was a positive correlation between the daily average temperature and O_3_ (*r* = 0.229) while negative correlations were found between the daily average temperature and the other three pollutants (*r*: −0.452 to −0.125); the daily average relative humidity was positively correlated with daily mean temperature (*r* = 0.153) while showed negative correlations with all four pollutants (*r*: −0.399 to −0.069).

**Table 2:** Summary of air quality and weather conditions during pregnancy of all participants.

Pollutants	Mean	Min	Max	Percentiles		
				5th	50th	95th
*Trimester 1*						
PM_2.5_ (μg/m^3^)	36.3	8.8	93.4	18.9	34.8	58.1
O_3_ (μg/m^3^)	54.1	16.7	96.8	32.9	54.0	75.8
NO_2_ (μg/m^3^)	34.3	5.0	88.7	13.5	33.5	58.8
SO_2_ (μg/m^3^)	14.2	4.3	58.4	6.7	12.4	27.4
Relative humidity (%)	78.5	53.9	90.5	69.1	79.2	85.5
Daily mean temperature (°C)	22.5	9.4	29.9	14.6	22.7	29.1
*Trimester 2*						
PM_2.5_ (μg/m^3^)	33.9	8.3	90.1	18.0	32.7	53.6
O_3_ (μg/m^3^)	54.4	16.9	97.6	34.6	54.5	74.0
NO_2_ (μg/m^3^)	33.2	8.5	83.6	13.2	32.8	57.3
SO_2_ (μg/m^3^)	13.1	4.5	54.6	6.8	11.8	23.9
Relative humidity (%)	79.1	57.0	90.3	70.6	79.6	85.6
Daily mean temperature (°C)	23.4	11.3	29.9	15.7	24.5	29.0
*Trimester 3*						
PM_2.5_ (μg/m^3^)	33.8	8.7	93.7	17.5	32.8	53.7
O_3_ (μg/m^3^)	55.2	14.2	105.7	34.5	55.1	75.1
NO_2_ (μg/m^3^)	33.3	7.0	96.8	13.2	32.8	57.1
SO_2_ (μg/m^3^)	12.6	4.0	65.1	6.8	11.6	22.6
Relative humidity (%)	78.8	43.9	95.3	70.0	79.2	85.7
Daily mean temperature (°C)	23.5	10.2	30.7	15.3	24.7	29.2

**Figure 1:**
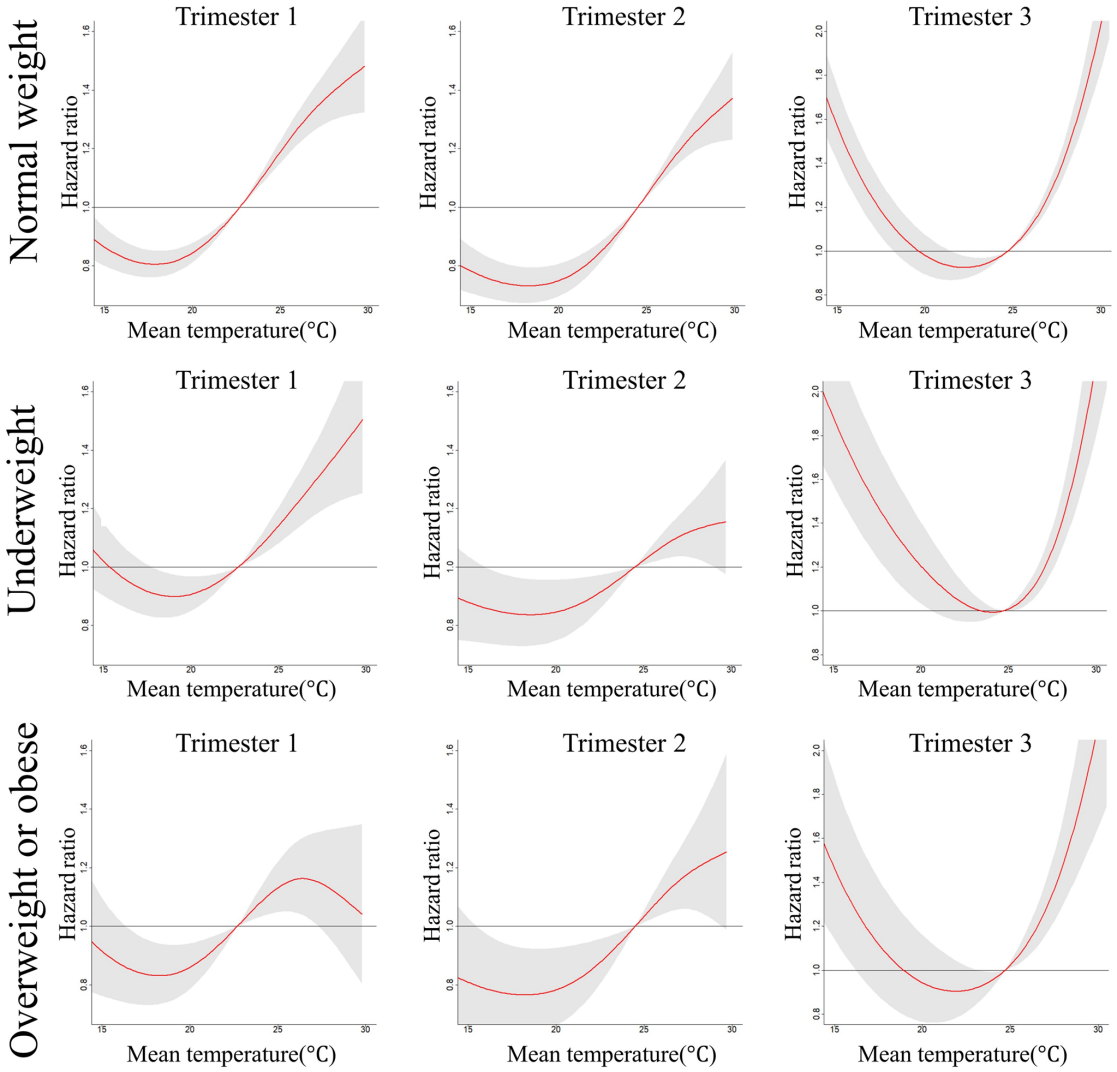
Hazard ratios (and 95% confidence intervals) of PTB associated with mean daily temperature during three trimesters of pregnancy across normal weight, underweight, overweight or obese women. Models were adjusted for characteristics of maternal age (<25 years, 25 ~ 34 years and ≥ 35 years), delivery mode (vaginal delivery and cesarean section), newborn gender (male and female), history of adverse pregnancy outcomes (PTB, miscarriage, induced abortion and stillbirth), active smoking, husband smoke and alcohol drinking status during early stage of pregnancy, season of delivery (spring, summer, fall and winter), mean of relative humidity and air pollutants (including PM_2.5_, O_3_, NO_2_ and SO_2_).

The smooth curves of the associations between mean daily temperature and PTB are provided in Figure 1. Overall, in all three subpopulations, the risk of PTB first decreased and then increased as the ambient temperature elevated, showing a J-shape or U-shape curve. Moreover, the HRs of cold exposure were below 1 only in the first and second trimesters, referring to a protective effect on PTB.

Table 3 further presents the HR estimates of the models at the three trimesters across different BMI levels. For all the BMI-related subgroups, corresponding with the results of our figures, the positive association between high-temperature exposure and the risk of PTB remained robust during all three trimesters, except that this effect failed to reach statistical significance for overweight or obese women in the first trimester. Meanwhile, the HRs were the highest in the third trimester during the entire pregnancy, especially for those overweight or obese women (HR:1.825; 95%CI:1.502 ~ 2.218). As a comparison, the low-temperature exposure significantly increased the risk of PTB only in the third trimester, while the results are differentiated during the first and second trimesters. For the normal weight, cold exposure was protective in the first (HR:0.881; 95%CI: 0.812 ~ 0.955) and second trimesters (HR: 0.763; 95%CI: 0.694 ~ 0.839). However, for women with abnormal BMI status, low temperature significantly reduced the risk of PTB only in the second trimester among those overweight or obese women. Overall, the largest effects were still detected in the third trimester during the whole pregnancy, extremely for those underweight women (HR: 1.825; 95%CI: 1.529 ~ 2.179).

**Table 3:** HRs (95% CIs) of the extreme temperature exposure (Model I^a^) for PTB according to maternal pre-pregnancy status of BMI.

BMI status	Gestational period	Low temperature	High temperature
Normal weight	1st trimester	0.881 (0.812, 0.955)	1.449 (1.317, 1.593)
	2nd trimester	0.763 (0.694, 0.839)	1.326 (1.222, 1.438)
	3rd trimester	1.512 (1.359, 1.682)	1.761 (1.621, 1.912)
Underweight	1st trimester	1.045 (0.917, 1.191)	1.449 (1.241, 1.692)
	2nd trimester	0.865 (0.741, 1.008)	1.146 (1.002, 1.311)
	3rd trimester	1.825 (1.529, 2.179)	1.784 (1.559, 2.042)
Overweight or Obesity	1st trimester	0.936 (0.771, 1.136)	1.077 (0.865, 1.341)
	2nd trimester	0.793 (0.636, 0.989)	1.233 (1.020, 1.491)
	3rd trimester	1.411 (1.104, 1.803)	1.825 (1.502, 2.218)

a Model I: adjusted for characteristics of maternal age (<25 years, 25 ~ 34 years and ≥ 35 years), delivery mode (vaginal delivery and cesarean section), newborn gender (male and female), history of adverse pregnancy outcomes (preterm birth, miscarriage, induced abortion and stillbirth), active smoking, husband smoke and alcohol drinking status during early stage of pregnancy, season of delivery (spring, summer, fall and winter), mean of relative humidity and air pollutants (including PM_2.5_, O_3_, NO_2_ and SO_2_).

Table 4 shows the estimated PTB burden attributable to extreme temperature in specific pregnancy periods. The largest attributable fractions were observed in the third trimester, for both the hot and cold exposure. Specifically, in the third trimester, cold exposure accounted for the greatest fraction of PTB (AF: 5.59, 95%CI: 3.58% ~ 7.98%) among underweight women, while hot exposure accounted for the greatest fraction of PTB (AF: 3.31, 95%CI: 2.01% ~ 4.88%) among overweight or obese women.

**Table 4:** Fraction and number of preterm births attributable to extreme temperatures.

Pre-pregnancy BMI categories	Trimester	Low temperature^a^		High temperature^a^	
		Attributable number	Attributable fraction (%)	Attributable number	Attributable fraction (%)
Normal weight	1	−72 (−114,-27)	−0.63 (−1,-0.24)	263 (186,347)	2.31 (1.63,3.05)
	2	−311 (−401,-211)	−2.74 (−3.54,-1.86)	223 (152,300)	2.08 (1.42,2.79)
	3	482 (338,642)	4.18 (2.93,5.56)	351 (286,420)	3.17 (2.58,3.79)
Underweight	1	4 (−8,17)	0.19 (−0.35,0.81)	49 (26,75)	2.26 (1.21,3.48)
	2	−28 (−54,2)	−1.33 (−2.56,0.08)	18 (0,39)	0.91 (0.01,1.93)
	3	121 (78,174)	5.59 (3.58,7.98)	66 (47,88)	3.14 (2.24,4.18)
Overweight or obese	1	−6 (−21,12)	−0.27 (−0.97,0.58)	8 (−15,37)	0.39 (−0.68,1.72)
	2	−43 (−76,-2)	−2.05 (−3.6,-0.11)	29 (3,62)	1.45 (0.12,3.05)
	3	61 (15,118)	2.78 (0.7,5.44)	69 (42,103)	3.31 (2.01,4.88)

a High temperatures were defined as temperatures above the 95th percentile, and low temperatures were defined as temperatures below the 5th percentile, based on the distribution of daily mean air temperature exposures throughout each trimester.

The results of our sensitivity analyses were similar to those of the main analyses (See Supplementary Tables S2 and S3). When changing the degrees of freedom for the temperature, the HRs of high-temperature exposure in the third trimester ranged from 1.649 to 1.761 for normal-weight women, 1.686 to 1.784 for those underweight, and 1.715 to 1.825 for those overweight or obese. Concerning the low temperature, the HRs in the third trimester ranged from 1.358 to 1.512 for normal-weight women, 1.588 to 1.825 for those underweight, and 1.319 to 1.411 for those overweight or obese. The results of various degrees of freedom for the relative humidity were similar. Moreover, compared with the results in the third trimester in Model I, the HRs of high-temperature exposure in Model II were 1.778 vs. 1.761 for normal-weight women, 1.782 vs. 1.784 for those underweight, and 1.869 vs. 1.825 for those overweight or obese; the corresponding HRs of low-temperature exposure were 1.520 vs. 1.512 for normal-weight women, 1.852 vs. 1.825 for those underweight, and 1.390 vs. 1.411 for those overweight or obese, respectively.

## 4. Discussion

The present large cohort study of 251,257 women in Guangdong, China showed that the risk of PTB rose with extreme temperature exposure during the third trimester, but decreased with cold exposure in the first and two trimesters. The association with low temperature was stronger for underweight women, while the association with high temperature was stronger for obese or overweight women. Our results add to the overall information on associations between extreme temperate exposures and PTB. The findings might also have implications for health workers, government officials, and the public to perform prevention strategies in the context of climate change.

There is mounting interest in the health impact of extreme weather conditions. Previous systematic reviews found a significant association between extreme temperature and PTB, especially high temperature (13, 28). Our results supported the prior finding that extreme heat elevated the risk of PTB during the whole pregnancy period. Currently, several plausible assumptions support the related mechanism, which attributes the high-temperature effect to the change in maternal hormone secretion and inflammation. To be specific, when heat exposure increases, maternal dehydration occurs, reducing the body fluid level and uterine blood flow (29–31). At the same time, the weakened maternal thermoregulation results in the release of cortisol, including increased secretion of oxytocin, prostaglandin, antidiuretic hormone, etc., which stimulates uterine contraction (32–34). Moreover, placental inflammation and dysfunction due to heat stress may also contribute to the increased risk of PTB. On the other hand, only a few studies focused on developing countries and involved the effects of cold exposure in their studies. Moreover, whether the impact of cold exposure reduces or increases the risk of PTB remains controversial at present. Overall, more studies reported the effect of cold exposure was harmful rather than protective, supporting a U-shaped association between ambient temperature and PTB (8, 35–37). Previous evidence indicated that vasoconstriction and blood viscosity increased in response to cold exposure, possibly reducing placental blood flow and thus affecting fetal growth (10). In our study, cold exposure was protective in the first and second trimesters, similar to the results of a few studies (11, 38). However, the possible physiological processes remain unclear. The variation of cold exposure impacts requires more research to fully clarify the phenomena.

Several studies investigated the critical exposure window for extreme temperature exposure but inconsistent exposure timeframes were identified. According to a review, most studies investigated the second and third trimester-effect of heat exposure (7). As for cold exposure, mostly the third trimester was reported (39). Our study found both the heat and cold exposure impacts were largest in the third trimester, which aligned with most studies. On another hand, the inconsistent results between studies were linked to the heterogeneity in the geographic locations, population characteristics and methodology. Specifically, varied definitions of exposure and timeframes might lead to differences in their findings.

To date, no literature has examined the different effects in each trimester across pre-pregnancy BMI subgroups, as in our study. In the third trimester, PTB risk from heat exposure was found to be the highest in women overweight and cold exposure in women underweight. Besides, we also observed a greater difference in HRs of PTB risk from cold exposure than heat exposure across the three BMI categories. It is reasonable to infer that pre-pregnancy BMI status may relate to one’s basic metabolism and thermoregulation efficiency, and thus those abnormal-weight women are physiologically less adaptive to temperature changes (40, 41). For example, overweight women could be resistant to cold exposure due to high-fat deposition and sensitive to hot exposure due to a low ratio of body surface area to volume. As for the greater HRs difference from cold exposure, one plausible explanation is that some adaptation measures to reduce the heat’s health impact are available, such as air conditioning and greenness space, while the heating system is not popular in southern China (42). In addition, the residents in southern China are less exposed to cold environments which may weaken their adaptation to cold exposure (43). Ambient temperature routine monitoring is necessary to guide the in-time adoption of prevention and control measures in society, including increased urban green and extreme temperature warnings to the public. Individual-level suggestions are also warranted to mitigate the disproportionate health burden from extreme temperature exposure, such as fewer maternal outdoor activities in heat waves and cold spells, especially for women with abnormal BMI levels.

Different from the HRs to show the associations, AN and AF aimed to appropriately quantify the disease burden caused by extreme temperature exposure. We found that the largest AF was in the third trimester and among women with abnormal weight, while the largest AN was still among the women with normal weight, for both the hot and cold exposure. In other words, relevant resources should be allocated to prevent the potentially increased PTB after extreme weather in the third trimester, especially for women with abnormal weight. Overall, the finding suggested that a substantial number of PTB should be reduced by controlling the extreme temperature exposure among pregnant women with normal weight.

Our study has the following limitations. First, other confounding factors might remain despite many covariates being adjusted in this study, such as maternal physical activity and occupation. Second, assigning the district-specific exposure estimates to each participant is approximate to the outdoor temperature rather than a real value of individual exposure, when considering the potential use of air conditioning or heating. Nevertheless, outdoor exposure is still meaningful for the guidance of future control strategies. Third, inaccurate exposure measurement may exist due to lacking information about maternal residence mobility and work address. Fourth, we excluded those without delivery outcomes and this would introduce selection bias in this study.

## 5. Conclusion

Exposure to either low temperature in the third trimester or high temperature during pregnancy could increase the risks of PTB. Overweight or obese women might be most susceptible to hot exposure, while the effect of low temperature was the strongest for underweight women. The findings would be useful to develop targeted and effective measures for vulnerable populations.

## Data availability statement

The datasets presented in this article are not readily available because they contained information that could compromise research participant privacy/consent. Requests to access the datasets should be directed to National Health Committee of China (NHCC) Key Laboratory of Male Reproduction and Genetics.

## Ethics statement

The studies involving human participants were reviewed and approved by the institutional review board of Guangdong Women and Children Hospital. The patients/participants provided their written informed consent to participate in this study.

## Author contributions

JQ: conceptualization, methodology, software, validation, writing - original draft, and writing - review and editing. ZL: conceptualization, formal analysis, funding acquisition, investigation, and writing - review and editing. JY: investigation, methodology, and validation. LX: investigation, and writing - review and editing. QX: methodology and software. XL: conceptualization, investigation, project administration, resources, supervision, and writing - review and editing. QZ: conceptualization, data curation, investigation, methodology, project administration, resources, supervision, and writing - review and editing. All authors contributed to the article and approved the submitted version.

## Funding

This work was partially supported by the Medical Science Foundation of Guangdong Province (C2020034).

## Conflict of interest

The authors declare that the research was conducted in the absence of any commercial or financial relationships that could be construed as a potential conflict of interest.

## Publisher’s note

All claims expressed in this article are solely those of the authors and do not necessarily represent those of their affiliated organizations, or those of the publisher, the editors and the reviewers. Any product that may be evaluated in this article, or claim that may be made by its manufacturer, is not guaranteed or endorsed by the publisher.
