# Supplementary material for: Extreme temperature exposure increases the risk of preterm birth in women with abnormal pre-pregnancy body mass index: a cohort study in a southern province of China
Source: Front Public Health. 2023 Jul 27;11:1156880. doi: 10.3389/fpubh.2023.1156880 (PMC10421663; doi:10.3389/fpubh.2023.1156880)
Supplement: Supplementary file 1 [file Table_1.doc]

**Table S1. Pearson’s correlation coefficients between meteorological variables and air pollutants during entire pregnancy of all participants.**

| Variables | PM_2.5_ | O_3_ | NO_2_ | SO_2_ | Temperature | Relative humidity |
| --- | --- | --- | --- | --- | --- | --- |
| PM_2.5_ | 1.000 | -0.110^*^ | 0.484^*^ | 0.657^*^ | -0.452^*^ | -0.399^*^ |
| O_3_ |  | 1.000 | -0.422^*^ | -0.054^*^ | 0.229^*^ | -0.069^*^ |
| NO_2_ |  |  | 1.000 | 0.133^*^ | -0.269^*^ | -0.315^*^ |
| SO_2_ |  |  |  | 1.000 | -0.125^*^ | -0.240^*^ |
| Temperature |  |  |  |  | 1.000 | 0.153^*^ |
| Relative humidity |  |  |  |  |  | 1.000 |

^*^P < 0.05.

Table S2. Sensitivity analysis for HRs (95% CIs) of the extreme temperature exposure (Model I^*^) for PTB according to maternal pre-pregnancy status of BMI.

| BMI status | Change in main model *^a^* | Low temperature | | |  | High temperature | | |
| --- | --- | --- | --- | --- | --- | --- | --- | --- |
|  |  | 1st trimester | 2nd trimester | 3rd trimester |  | 1st trimester | 2nd trimester | 3rd trimester |
| Normal weight | df for temperature | | | |  |  |  |  |
|  | 2 | 0.894 (0.824, 0.971) | 0.805 (0.734, 0.884) | 1.491 (1.360, 1.635) |  | 1.556 (1.420, 1.706) | 1.400 (1.297, 1.510) | 1.737 (1.602, 1.883) |
|  | 3 | 0.881 (0.812, 0.955) | 0.763 (0.694, 0.839) | 1.512 (1.359, 1.682) |  | 1.449 (1.317, 1.593) | 1.326 (1.222, 1.438) | 1.761 (1.621, 1.912) |
|  | 4 | 0.920 (0.846, 1.000) | 0.766 (0.696, 0.843) | 1.358 (1.226, 1.505) |  | 1.503 (1.363, 1.657) | 1.326 (1.223, 1.438) | 1.649 (1.524, 1.784) |
|  | df for RH |  |  |  |  |  |  |  |
|  | 2 | 0.881 (0.812, 0.955) | 0.765 (0.696, 0.842) | 1.506 (1.354, 1.675) |  | 1.449 (1.317, 1.593) | 1.324 (1.220, 1.436) | 1.768 (1.628, 1.920) |
|  | 3 | 0.881 (0.812, 0.955) | 0.763 (0.694, 0.839) | 1.512 (1.359, 1.682) |  | 1.449 (1.317, 1.593) | 1.326 (1.222, 1.438) | 1.761 (1.621, 1.912) |
|  | 4 | 0.881 (0.812, 0.955) | 0.765 (0.695, 0.841) | 1.503 (1.351, 1.672) |  | 1.451 (1.320, 1.596) | 1.330 (1.226, 1.443) | 1.739 (1.601, 1.889) |
| Underweight | df for temperature | | | |  |  |  |  |
|  | 2 | 1.058 (0.927, 1.207) | 0.899 (0.774, 1.043) | 1.588 (1.371, 1.838) |  | 1.510 (1.299, 1.755) | 1.188 (1.049, 1.347) | 1.686 (1.477, 1.924) |
|  | 3 | 1.045 (0.917, 1.191) | 0.865 (0.741, 1.008) | 1.825 (1.529, 2.179) |  | 1.449 (1.241, 1.692) | 1.146 (1.002, 1.311) | 1.784 (1.559, 2.042) |
|  | 4 | 1.081 (0.943, 1.239) | 0.854 (0.732, 0.997) | 1.684 (1.420, 1.996) |  | 1.488 (1.269, 1.745) | 1.140 (0.998, 1.302) | 1.715 (1.507, 1.951) |
|  | df for RH |  |  |  |  |  |  |  |
|  | 2 | 1.045(0.917, 1.190) | 0.867 (0.742, 1.012) | 1.813 (1.519, 2.164) |  | 1.449 (1.241, 1.692) | 1.138 (0.996, 1.302) | 1.802 (1.575, 2.061) |
|  | 3 | 1.045 (0.917, 1.191) | 0.865 (0.741, 1.008) | 1.825 (1.529, 2.179) |  | 1.449 (1.241, 1.692) | 1.146 (1.002, 1.311) | 1.784 (1.559, 2.042) |
|  | 4 | 1.046 (0.918, 1.192) | 0.867 (0.742, 1.012) | 1.819 (1.524, 2.171) |  | 1.463 (1.253, 1.710) | 1.143 (1.000, 1.308) | 1.755 (1.534, 2.008) |
| Overweight or Obesity | df for temperature | | | |  |  |  |  |
|  | 2 | 0.944 (0.774, 1.152) | 0.824 (0.661, 1.027) | 1.399 (1.126, 1.740) |  | 1.186 (0.959, 1.466) | 1.297 (1.086, 1.548) | 1.806 (1.492, 2.188) |
|  | 3 | 0.936 (0.771, 1.136) | 0.793 (0.636, 0.989) | 1.411 (1.104, 1.803) |  | 1.077 (0.865, 1.341) | 1.233 (1.020, 1.491) | 1.825 (1.502, 2.218) |
|  | 4 | 0.983 (0.804, 1.201) | 0.799 (0.639, 0.998) | 1.319 (1.040, 1.673) |  | 1.123 (0.896, 1.408) | 1.233 (1.020, 1.491) | 1.715 (1.421, 2.069) |
|  | df for RH |  |  |  |  |  |  |  |
|  | 2 | 0.936 (0.771, 1.137) | 0.793 (0.635, 0.992) | 1.417 (1.109, 1.810) |  | 1.076 (0.865, 1.340) | 1.230 (1.017, 1.487) | 1.816 (1.495, 2.206) |
|  | 3 | 0.936 (0.771, 1.136) | 0.793 (0.636, 0.989) | 1.411 (1.104, 1.803) |  | 1.077 (0.865, 1.341) | 1.233 (1.020, 1.491) | 1.825 (1.502, 2.218) |
|  | 4 | 0.936 (0.771, 1.137) | 0.793 (0.634, 0.991) | 1.396 (1.092, 1.786) |  | 1.076 (0.864, 1.339) | 1.250 (1.033, 1.512) | 1.811 (1.491, 2.200) |

*^a^* The main Cox proportional hazards model for mean concentrations of each trimester, 3 dfs for mean ambient temperature, and 3 dfs for mean relative humidity.

^*^Model I: adjusted for characteristics of maternal age (<25 years, 25~34 years and ≥35 years), delivery mode (vaginal delivery and cesarean section), newborn gender (male and female), history of adverse pregnancy outcomes (preterm birth, miscarriage, induced abortion and stillbirth), active smoking, husband smoke and alcohol drinking status during early stage of pregnancy, season of delivery (spring, summer, fall and winter), mean of relative humidity and air pollutants (including PM_2.5_, O_3_, NO_2_ and SO_2_).

Table S3. HRs (95% CIs) of the extreme temperature exposure (Model Ⅱ^*^) for PTB according to maternal pre-pregnancy status of BMI.

| BMI status | Gestational period | Low temperature | High temperature |
| --- | --- | --- | --- |
| Normal weight | 1st trimester | 0.884 (0.817, 0.958) | 1.466 (1.334, 1.610) |
|  | 2nd trimester | 0.764 (0.696, 0.839) | 1.326 (1.223, 1.437) |
|  | 3rd trimester | 1.520 (1.368, 1.688) | 1.778 (1.639, 1.929) |
| Underweight | 1st trimester | 1.058 (0.930, 1.202) | 1.437 (1.233, 1.675) |
|  | 2nd trimester | 0.850 (0.731, 0.989) | 1.138 (0.997, 1.300) |
|  | 3rd trimester | 1.852 (1.555, 2.205) | 1.782 (1.560, 2.035) |
| Overweight or Obesity | 1st trimester | 0.924 (0.763, 1.120) | 1.129 (0.908, 1.402) |
|  | 2nd trimester | 0.757 (0.608, 0.942) | 1.232 (1.020, 1.488) |
|  | 3rd trimester | 1.390 (1.091, 1.773) | 1.869 (1.542, 2.266) |

^*^Model Ⅱ: adjusted for season of delivery (spring, summer, fall and winter), mean of relative humidity and air pollutants (including PM_2.5_, O_3_, NO_2_ and SO_2_).
